# 2022 mLife reviewer acknowledgments

**DOI:** 10.1002/mlf2.12057

**Published:** 2023-02-06

mLife would like to thank all of the below reviewers for their great support and efforts in 2022.

Koichiro Awai

Gustavo Caetano‐Anolles

Agamemnon J. Carpousis

Jasper Fuk‐Woo Chan

Wenli Chen

Haiyan Chu

Li Cui

Lei Dai

Chittaranjan Das

Ye Deng

Xianding Deng

Mark R. Denison

Xiuzhu Dong

Arnold J. M. Driessen

Kai Feng

Greg Fournier

Kudo Fumitaka

Haichun Gao

Ramon Gonzalez

Jeffrey Gralnick

Tingyue Gu

Ji‐Dong Gu

Xue Guo

Cedric Jasper Hahn

Anil Haritash

Zhili He

Qiang He

Youcai Hu

Baolan Hu

Zhengshuang Hua

Wei Huang

Alexander Idnurm

William P Inskeep

Yoshizumi Ishino

Dieter Jahn

Lin Jiang

Daniel Kearns

Ralf Koebnik

Mart Krupovic

Jialiang Kuang

Ming Li

Wen‐Jun Li

Meng Li

Ning Ling

Yuguang Liu

Yang Liu

Tong‐Bao Liu

Xi‐Peng Liu

Frank E. Löffler

Allison Lopatkin

Huiqiang Lou

Ricardo O. Louro

Yao Lu

Zhao‐Qing Luo

Zhenmei Lv

Saurabh Mahajan

Yuxin Mao

Bernard de Massy

Shawn McGlynn

Marc Mussmann

Matthew J. Neale

Hayley J. Newton

Kang Ning

Hongyu Ou

Catarina M. Paquete

Jingjing Peng

Guoliang Qian

Wei Qian

Cheng‐Feng Qin

Jean‐Baptiste Raina

Zhiming Rao

Peter Redder

Gemma Reguera

Zhigang Ren

Minglei Ren

Jason Ren

Amelia Rotaru

Annette R. Rowe

Jue Ruan

Zongze Shao

Qunxin She

Xihui Shen

Yulong Shen

Liang Shi

Zhou Shi

Shuobo Shi

Fuping Song

Yizhi Song

Jun Sun

Yanni Sun

Clifford Swanson

Zhiyao Tang

Armando Tejeda

Jing Tian

Judy Wall

Shanquan Wang

Jiawei Wang

Wei Wang

Fengping Wang

Yue Wang

Xiaoxue Wang

Kenneth Wasmund

William B Whitman

Xiao‐Lei Wu

Weihui Wu

Ai‐Bo Wu

Hao Wu

Zhenjiang Xu

Lei Xu

Ping Xu

Jian Xu

Chaoyang Xue

Zaisheng Yan

Aixin Yan

Xinxing Yang

Yunfeng Yang

Huabing Yin

You Yu

Mengting Yuan

Chong Zhang

Lin Zhang

Liangran Zhang

Dayi Zhang

Jing‐Ren Zhang

Xiaohua Zhang

Zheng Zhang

Lin Zhang

Chenhong Zhang

Xilin Zhao

George Zhou

Shu Zhu

Yongqun Zhu

